# Lacticaseibacillus rhamnosus Infective Endocarditis Complicated by Multifocal Mycotic Aneurysms in an Immunocompetent Patient: A Case Report

**DOI:** 10.7759/cureus.68056

**Published:** 2024-08-28

**Authors:** Taichi Ito, Michihiro Okuyama, Yu Takahashi, Kiyofumi Ohkusu, Kyoko Yokota

**Affiliations:** 1 Department of Respirology, Kagawa Prefectural Central Hospital, Takamatsu, JPN; 2 Department of Cardiovascular Surgery, Kagawa Prefectural Central Hospital, Takamatsu, JPN; 3 Department of Neurosurgery, Kagawa Prefectural Central Hospital, Takamatsu, JPN; 4 Department of Microbiology, Tokyo Medical University, Tokyo, JPN; 5 Department of Infectious Diseases, Kagawa Prefectural Central Hospital, Takamatsu, JPN

**Keywords:** lactobacillaceae, probiotics, mycotic aneurysms, lacticaseibacillus rhamnosus, infective endocarditis

## Abstract

*Lactobacillaceae* are generally considered low-pathogenicity organisms but can occasionally cause severe infections. We report a severe case of infective endocarditis caused by *Lacticaseibacillus rhamnosus *complicated by multifocal mycotic aneurysms in an immunocompetent patient who consumed probiotic drinks daily. He had a history of hypertension and aortic valve regurgitation.After starting antimicrobial treatment, the patient had rapid disease progression and required emergency surgery to prevent the rupture of two abdominal aneurysms. He subsequently experienced a rupture of an intracranial aneurysm, leading to a subarachnoid and intraventricular hemorrhage. This case highlights the potential severity of *Lactobacillaceae* infections, even in immunocompetent individuals with daily probiotic consumption. Interest in the health benefits of probiotics has grown in recent years, leading to increased global demand. The number of reported cases of *Lactobacillaceae* infections has also increased. When using probiotics, both the potential benefits and risks need to be considered, especially in susceptible individuals with predisposing conditions.

## Introduction

*Lactobacillaceae* are widely distributed members of the human commensal flora, commonly found in the oral cavity, gastrointestinal tract, and urogenital system. *Lactobacillaceae* is one of the representative bacterial families used in probiotics, which are administered to maintain a favorable state of the gut microbiota, thereby normalizing intestinal and immune functions. Their efficacy has been suggested through meta-analyses for various disease categories [1]*.*

Although *Lactobacillaceae* are generally recognized as low-pathogenicity organisms in humans, certain species can occasionally cause infections in individuals with specific risk factors, such as an immunocompromised state or certain anatomical abnormalities. *Lactobacillus* species have been reported to cause infective endocarditis (IE) in susceptible individuals, particularly those who are immunocompromised or have valvular heart disease [2].

In this paper, we report a severe case of infective endocarditis caused by *Lacticaseibacillus rhamnosus* complicated by multifocal mycotic aneurysms. To our knowledge, no similar cases have been reported previously. We believe that our case provides important lessons regarding the characteristics of infections caused by *Lactobacillaceae* and the use of probiotics in susceptible individuals. Herein, we present our case and discuss its clinical implications.

## Case presentation

**Figure 1:**
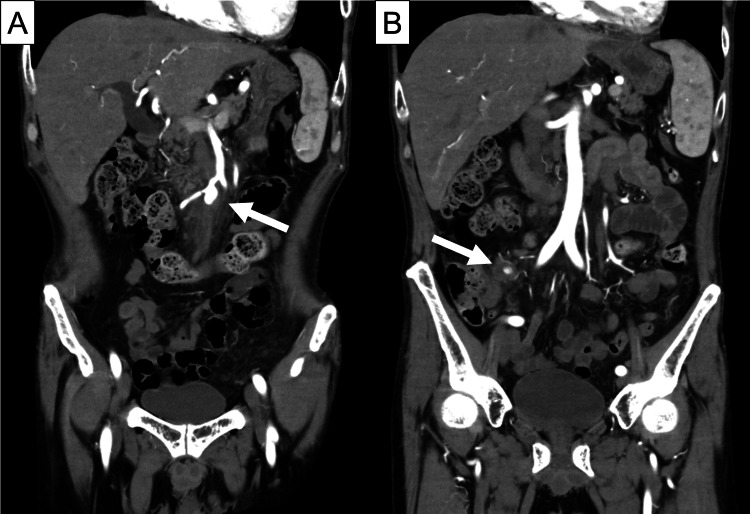
Abdominal contrast-enhanced computed tomography (CT) on day 0. Aneurysms of the ileal artery (A, arrow) and ileocolic artery (B, arrow).

A man in his 50s with a two-month history of fever, fatigue, arthralgia, myalgia, anorexia, and weight loss of 15 kg presented to the emergency room with worsening abdominal pain for this one week. His medical history included hypertension and aortic valve regurgitation. Abdominal computed tomography angiography (CTA) revealed aneurysms arising from the origin of the ileal and ileocolic arteries (Figure 1). The patient was transferred to a tertiary care hospital for further evaluation of the intra-abdominal arterial lesions.

On examination, he was alert and oriented. Vital signs were as follows: temperature 37.4°C, heart rate 101 beats per minute, blood pressure 112/76 mmHg, and respiratory rate 18 breaths per minute. Oral examination revealed poor oral hygiene. Cardiac auscultation revealed a pansystolic murmur at the apex. Abdominal examination showed a soft, non-tender abdomen without guarding or rebound tenderness. He had a 15-pack-year smoking history and no alcohol use, and no history of HIV infection or injection drug use. He reported daily consumption of several commercial probiotic drinks. 

Laboratory tests showed leukocytosis (white blood cell count 16700 cells/µL) and elevated C-reactive protein level (18.7 mg/dL). Liver function tests, renal function tests, and coagulation profiles were unremarkable. Biochemical analysis revealed no abnormalities suggestive of diabetes mellitus or dyslipidemia. Owing to the intra-abdominal arterial lesions and the two-month history of fever and weight loss, the patient was admitted with suspected vasculitis.

On the second day after admission, transthoracic echocardiography revealed a 9 mm vegetation on the aortic valve and an 8 mm vegetation on the mitral valve. Severe regurgitation was observed in both valves. Antimicrobial therapy with ceftriaxone and vancomycin was initiated for clinically diagnosed IE. Despite severe regurgitation, hemodynamic stability was maintained without evidence of congestive heart failure; consequently, elective surgery was planned. On the third day after admission, blood cultures grew Gram-positive bacilli, which were identified as *Lacticaseibacillus rhamnosus* (formerly *Lactobacillus rhamnosus*) using Matrix-assisted laser desorption-ionization time-of-flight mass spectrometry (MALDI-TOF MS) and subsequently confirmed using 16S ribosomal DNA sequencing. Based on culture results, the antibiotics were switched to ampicillin and gentamicin. Blood cultures were negative on day nine of the admission.

**Figure 2:**
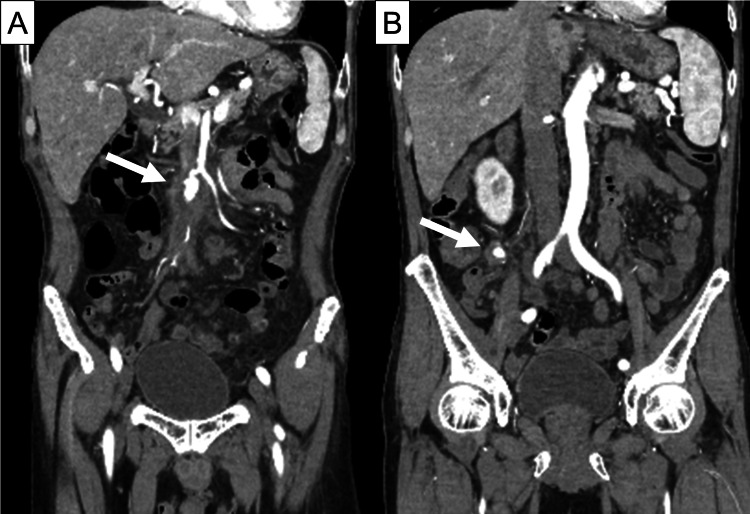
Abdominal contras-enhanced computed tomography (CT) on day 9. (A) Enlargement of the ileal artery aneurysm with surrounding low-density area (arrow), suggesting progression of the infection and necessitating urgent surgical intervention. (B) Mild enlargement of the ileocolic artery aneurysm (arrow) similarly indicated progression of the infection.

On day nine of the admission, the patient developed worsening abdominal pain. Abdominal CTA showed enlargement of the aneurysms (Figure 2). Based on the diagnosis of an impending aneurysm rupture, emergency surgery was performed. Intraoperative findings showed pus surrounding the aneurysms, but cultures were negative. Both aneurysms were resected, followed by vascular reconstruction and ileocecal resection. Histopathological findings of the resected aneurysms were consistent with mycotic aneurysms. Similarly, histopathological analysis of the resected intestine and mesentery showed signs of inflammation, but no significant findings such as perforation were observed. Antimicrobial therapy was continued postoperatively.

On day 36 of admission, the patient underwent aortic valve replacement and mitral valve repair. Both the aortic and mitral valves were perforated. Histopathological findings were consistent with those of healed IE. Cultures of the valves and vegetation were negative.

**Figure 3:**
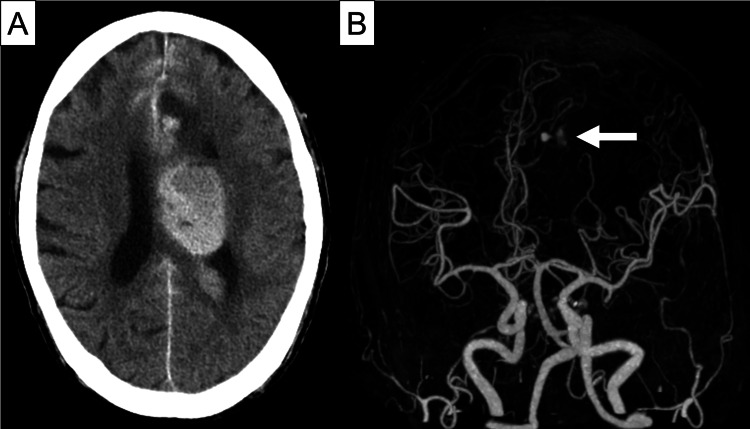
Head computed tomography (CT) and CT angiography (CTA) on day 52. (A) Head CT showing subarachnoid and intraventricular hemorrhage, necessitating immediate external ventricular drainage to manage intracranial pressure. (B) Head CTA revealing an aneurysm with extravasation of the peripheral segment of the left anterior cerebral artery (arrow), indicating the urgent need for intervention to treat the aneurysm and prevent further bleeding.

He continued to receive antibiotic treatment after surgery. However, on day 52 of admission (day 16 post-surgery), he experienced a transient loss of consciousness and right hemiparesis. CT of the head revealed subarachnoid and intraventricular hemorrhages (Figure 3A). CT angiography revealed an aneurysm with extravasation in the peripheral segment of the left anterior cerebral artery (Figure 3B). After consultation with neurosurgery, emergency coil embolization of the aneurysm and external ventricular drainage were performed. Blood and cerebrospinal fluid cultures were obtained, and the results were negative. Although these interventions were lifesaving, the patient was left with significant neurological deficits. Antimicrobial therapy with ampicillin and gentamicin was continued for four weeks after the endovascular procedure, resulting in a total treatment duration of 80 days from the start of therapy. The patient was subsequently transferred to a rehabilitation facility.

## Discussion

We report a severe case of IE involving both the aortic and mitral valves caused by *Lacticaseibacillus rhamnosus*, complicated by multifocal mycotic aneurysms in the intra-abdominal and intracranial arteries. To our knowledge, this is the first reported case of IE caused by *L. rhamnosus* complicated by ruptured multifocal mycotic aneurysms. Although reports of vascular infections caused by *L. rhamnosus* are relatively uncommon, similar cases of severe infection have been documented in individuals without significant underlying diseases [2]. When consuming probiotic products containing lactobacilli, careful consideration is needed regarding their potential benefits and risks.

Although *Lactobacillaceae* are generally considered to have low pathogenicity in humans, certain species can cause various infections, including bacteremia, intra-abdominal abscesses, oral infections, intrauterine infections, and IE. Previous studies have identified several factors associated with *Lactobacillus* infections, including an immunocompromised state, malignancies, anatomical abnormalities, recent dental or genital procedures, and excessive consumption of probiotic products [3].

Several studies have described the characteristics of *Lactobacillus* IE. Ioannou et al. [2] analyzed 82 cases and found a median patient age of 53.7 years, with 34 cases (42%) having poor oral hygiene or a history of dental procedures, and 14 of 81 cases (17%) having a history of probiotic use. Campagne et al. [4] identified valvular heart disease, valve prostheses, dental procedures, and probiotic use as common background factors in patients with *Lactobacillus* IE. The reported mortality rate of *Lactobacillus* IE ranges from 10% to 12.2% [3,4]. Notably, the duration of symptoms, such as fever, weight loss, and fatigue, before diagnosis varies widely from two days [5] to nine months [4]. This wide range suggests that *Lactobacillus* IE may have a prolonged course, possibly starting several months before diagnosis. Our patient developed symptoms two months before diagnosis. He had a history of aortic valve regurgitation and poor oral hygiene, which have previously been reported as risk factors for *Lactobacillu*s IE. No other common risk factors were identified. Notably, although the patient regularly consumed probiotic drinks, his daily intake ranged from 200 to 300 ml, which did not significantly exceed the daily recommended intake by the manufacturers of these products.

Additionally, a notable feature in our case was the development of multifocal mycotic aneurysms involving the intra-abdominal and intracranial arteries. To our knowledge, no previous reports have described *Lactobacillus* IE complicated by extensive multifocal mycotic aneurysms. Agrawal et al. [6] reported a fatal case of *Lactobacillus* IE following transcatheter aortic valve replacement complicated by rupture of a cerebral aneurysm. Sturdee et al. [7] described a case of mycotic aneurysm in the splenic artery caused by *Lactobacillus* species without endocarditis. According to their report, the patient experienced a cerebral hemorrhage five months after treatment, suggesting potential long-term vascular sequelae of *Lactobacillus* infection. Terao et al. [8] reported a case of IE and an intracranial mycotic aneurysm caused by *L. rhamnosus* in an individual with no known history of valvular heart disease or immunodeficiency. This case was associated with excessive yogurt consumption (>1 L/day), highlighting the potential risks of probiotic overuse even in otherwise healthy individuals.

Growing global interest in health promotion and preventive medicine has led to an increased focus on probiotics, particularly during the COVID-19 pandemic. The global probiotic market was estimated at 68.56 billion USD in 2022 and is projected to expand to 133.92 billion USD by 2030 [9]. Although the health benefits of probiotics are gaining worldwide attention, their potential negative effects must also be considered. Costa et al. [10] reported the factors associated with infections following probiotic use, including underlying conditions such as immunosuppression, malignancies, and the use of central venous catheters. Sada et al. [11] reported cases of bacteremia caused by *Clostridium butyricum* in immunocompromised patients using probiotics containing this organism. Their findings emphasize the need for careful consideration of the balance between the potential risks and benefits of probiotic use in immunocompromised individuals. Recent reports have shown an increase in infections caused by *Lactobacillus* species, which is thought to be associated with the increase in the consumption of probiotics in recent years [12]. Based on these findings, we believe a careful assessment of both the benefits and risks of probiotic use is necessary, even in apparently healthy individuals.

In this paper, we described a case of *Lacticaseibacillus rhamnosus* IE with multifocal mycotic aneurysms in an immunocompetent patient with valvular heart disease and poor oral hygiene. This case demonstrates that *Lactobacillaceae* can cause severe infections, even in individuals without apparent immunodeficiency. Notably, our patient developed a severe infection that may be associated with daily consumption of commercial probiotic drinks, raising important questions about the safety of regular probiotic use in individuals with predisposing factors such as valvular heart disease and poor oral hygiene. Our case suggests the need for careful consideration of probiotic use, including commercial products, even in typical doses, in individuals with risk factors for *Lactobacillus* infections.

Evidence regarding the benefits of probiotics has been accumulated, while large-scale studies examining their potential negative effects are limited. Our case suggests that attention may need to be paid to the potential negative effects of probiotics in certain patient populations. We believe that further research is necessary to investigate the potential negative effects of probiotics and their associated risk factors. Additionally, studies are needed to establish guidelines for safe probiotic use in different patient populations, particularly in those with underlying risk factors for infection.

## Conclusions

This case of *L. rhamnosus* IE with multifocal mycotic aneurysms in an immunocompetent patient highlights the potential severity of *Lactobacillus* infections and the importance of aggressive management of IE complications. Furthermore, careful consideration is required to identify individual risk factors and weigh both the benefits and potential risks of probiotic use, including commercial products.
